# Pre-Service Teachers’ GenAI Anxiety, Technology Self-Efficacy, and TPACK: Their Structural Relations with Behavioral Intention to Design GenAI-Assisted Teaching

**DOI:** 10.3390/bs14050373

**Published:** 2024-04-29

**Authors:** Kai Wang, Qianqian Ruan, Xiaoxuan Zhang, Chunhua Fu, Boyuan Duan

**Affiliations:** 1 Center for Teacher Education Research, Beijing Normal University, Beijing 100091, China; wangkai1991@bnu.edu.cn; 2 School of Education, Minzu University of China, Beijing 100081, China; fuchunhua161618@163.com (C.F.); 21301412@muc.edu.cn (B.D.); 3 School of Education, Central China Normal University, Wuhan 430070, China; xiaoxuan19990705@163.com

**Keywords:** generative artificial intelligence, pre-service teachers, the UTAUT model, TPACK, anxiety

## Abstract

Generative artificial intelligence (GenAI) has taken educational settings by storm in the past year due to its transformative ability to impact school education. It is crucial to investigate pre-service teachers’ viewpoints to effectively incorporate GenAI tools into their instructional practices. Data gathered from 606 pre-service teachers were analyzed to explore the predictors of behavioral intention to design Gen AI-assisted teaching. Based on the Unified Theory of Acceptance and Use of Technology (UTAUT) model, this research integrates multiple variables such as Technological Pedagogical Content Knowledge (TPACK), GenAI anxiety, and technology self-efficacy. Our findings revealed that GenAI anxiety, social influence, and performance expectancy significantly predicted pre-service teachers’ behavioral intention to design GenAI-assisted teaching. However, effort expectancy and facilitating conditions were not statistically associated with pre-service teachers’ behavioral intentions. These findings offer significant insights into the intricate relationships between predictors that influence pre-service teachers’ perspectives and intentions regarding GenAI technology.

## 1. Introduction

Artificial intelligence (AI) has become a significant and transformative factor across diverse academic disciplines and industries, including research, teaching, and business [1,2]. Since its inception, AI has experienced substantial growth, particularly with advances in artificial neural networks (ANN) and deep learning (DL), allowing for a notable improvement in generative artificial intelligence (GenAI) [3]. GenAI is a technology that generates various forms of human-like content, such as text, images, videos, and audio, by effectively responding to complex prompts expressed in natural language text [3,4]. It is currently pushing the boundaries of education and causing a revolution in its practice [5]. GenAI tools, such as ChatGPT, can provide valuable support for students by giving them helpful feedback and improving their interactive and adaptable learning experience [5,6]. Teachers can also benefit from it in various ways, including the implementation of effective pedagogical approaches, the facilitation of course content production, the improvement of evaluation methods, and the enhancement of management efficiency [7,8,9]. As Chen et al. [10] pointed out, with the help of GenAI technology, teachers can effectively reduce their workload and truly focus on urgent matters.

Despite the potential benefits of integrating GenAI into educational practices, the incorporation may encounter certain challenges [11,12,13]. Research suggests that educators have not fully embraced technology due to skepticism and reluctance towards its use [14,15,16]. The integration of new technologies into teachers’ instructional methods may face obstacles due to a range of challenges, including external factors such as insufficient support, a lack of essential technical resources, and curricular constraints, as well as internal factors like insufficient technical skills or low self-efficacy [17,18,19]. Research has also revealed that some educators prefer to use familiar resources and instructional methodologies and are frequently anxious about new technology, thus leading to their hesitance in technology adoption [20]. These external and internal factors may also hinder teachers from implementing GenAI technologies.

Given the current rise of GenAI technology in education and the existing challenges, it is essential to fully comprehend pre-service teachers’ viewpoints and willingness towards it [21], thus helping ensure that they are well-prepared to utilize GenAI in assisting their teaching. However, comprehensive research on the factors influencing teachers’ adoption of GenAI remains limited [22]. Specifically, research on pre-service teachers’ attitudes towards its utilization has only just emerged [23]. Researchers also pointed out that pre-service teachers face difficulties in properly adopting GenAI to improve their pedagogical literacy [24]. Consequently, the objective of this study is to investigate the perspectives of pre-service teachers regarding the integration of GenAI into their teaching methods and identify both internal and external factors impacting their acceptance. The Unified Theory of Acceptance and Use of Technology (UTAUT) model was utilized as a theoretical framework to examine the external factors. Simultaneously, this study incorporated the Technology Pedagogical and Content Knowledge (TPACK) framework, along with constructs like technology self-efficacy and GenAI anxiety, to clarify the internal determinants that influence pre-service teachers’ perspectives on GenAI.

The UTAUT model is particularly valuable for examining how eternal factors, such as its usefulness, simplicity, and support from organizations and social networks, impact individuals’ inclination toward technology adoption [25]. TPACK is a frequently used framework to assist teachers in efficiently incorporating technology into their instructional methods, serving as a crucial supplement to the UTAUT model [26]. The evaluation of GenAI anxiety and technology self-efficacy includes both the emotional and cognitive responses to GenAI when using it. By adopting a thorough consideration of internal and external influences, this study may offer an in-depth understanding of the elements that impact pre-service teachers’ acceptance of GenAI, which is essential for developing targeted and effective strategies to integrate technology in educational contexts.

## 2. Literature Review

### 2.1. AI and Generative AI in Education

As more scholars and educational organizations investigate the potential advantages of AI, the application of AI in education (AIED) has gained traction [27]. AIED systems can manifest in various formats and can be categorized into learner-facing systems (e.g., Intelligent Tutoring Systems), educator-facing systems (e.g., automated grading support), and institutional support (e.g., identifying students at risk of attrition) [28]. The current research on AIED primarily investigates the utilization of AI in the implementation of teaching and learning, including the design, application, and evaluation of teaching tools, teaching models, teaching strategies and frameworks, ethical implications, and teacher AI literacy [29].

The emergence of GenAI has accelerated its integration into education practices. This has resulted in changes to the learning methods of students, the teaching and assessment practices of teachers, and the policy modifications of educational institutions [30,31]. GenAI tools, like ChatGPT, assist teachers in generating learning quizzes [32], developing teaching units using the “5Es model” (engage, explore, explain, elaborate, and evaluate), and providing scaffold for students who are facing difficulties [33]. Another example is that intelligent tutoring systems can support learning by teaching course content, managing learning resources, assessing student strengths and weaknesses, offering automatic feedback, and promoting cooperation among learners [34].

The immense potential of generative AI techniques and tools in the field of education has been widely acknowledged and has already become an integral part of modern life [35]. Therefore, it is crucial to explore methods of integrating them into education. Teachers are essential stakeholders in the process of GenAI-assisted teaching and learning. To effectively integrate GenAI into their teaching practices, pre-service teachers—as future educators—need to take into account their viewpoints and expectations. Nevertheless, there remains a dearth of adequate empirical investigation delving into their perspectives on GenAI tools [36]. In order to deliver effective professional learning that reinforces pre-service teachers’ belief systems, the aim of this study was to investigate the factors that influence their perspectives and willingness to use GenAI through a quantitative analysis.

### 2.2. Hypotheses Development

#### 2.2.1. The UTAUT Model

The Unified Theory of Acceptance and Use of Technology (UTAUT) model is a well-established framework for investigating how individuals’ perceptions about the eternal factors impact their intention to use technology [37]. Originally developed by Venkatesh et al. [38], it combines elements from eight established theories, including the Theory of Reasoned Action, the Motivational Model, and the Model of PC Utilization [39]. This comprehensive model specifically emphasizes four key elements—performance expectancy, effort expectancy, social influence, and facilitating conditions—to explain the factors influencing users’ acceptance and utilization behavior towards technologies [38,40]. The UTAUT model, renowned for its high predictive accuracy, has been found to be approximately 70% effective in predicting technology acceptance [41], establishing it as a key theory in the field of technology adoption [42].

Effort expectancy refers to the perceived ease of using a system [38]. In this research, it is defined as the perception of simplicity in adopting GenAI technologies to assist teaching. Consistent with the UTAUT model, numerous studies have verified that effort expectancy has a substantial impact on teachers’ behavioral intention to adopt technologies [43,44]. However, Alotumi’s [40] investigation of graduate students’ intention to use Google Classes suggested that this relationship may not always hold true, as effort expectancy failed to predict behavioral intention. Hence, further exploration is necessary for testing the effectiveness of effort expectancy in shaping individuals’ behavioral intentions, particularly within the realm of GenAI adoption.

Social influence refers to individuals’ perceptions of influential others supporting the utilization of a new technology system [38]. In this research, the term refers to the extent to which pre-service teachers perceive that others’ opinions about GenAI impact their decision to adopt it. Venkatesh [45] has noted that opinions from friends and family can greatly shape a user’s inclination towards embracing novel technologies. Empirical studies have also consistently demonstrated the influential impact of social influence on an individual’s inclination towards utilizing technologies [46,47,48]. However, some studies have found contrasting results where the impact of social influence on teachers’ intentions to use technologies was found to be insignificant [49,50].

Facilitating conditions is the degree to which a user perceives adequate organizational and technical support for utilizing technologies [38]. This study defines this term as pre-service teachers’ perceived assistance from their organizations, such as hardware and software support, administrative endorsement, skill training, and technical guidance [51]. Notably, Fathi and Ebadi [52] verified that technical support was the most influential factor affecting pre-service teachers’ adoption of technology. Studies conducted by Kim and Lee [53], Ning, Yang, Zhu, Bayarmaa, and Ma [39], and Wong [54] have also consistently proven the significant impact of these facilitating conditions on teachers’ intentions to use technology in their teaching practices and also emphasized the necessity of establishing a sound supportive infrastructure to ensure effective technology integration in education.

Performance expectancy refers to individuals’ perception of the potential benefits that a certain technology can bring to their job performance enhancement [38]. This study defines it as pre-service teachers’ belief in the effectiveness of GenAI in improving their teaching performance. Recent research has also found that performance expectancy serves as a significant predictor of technology adoption in educational settings [44,55], and it is even identified as the primary factor influencing individuals’ intentions in the UTAUT model [56].

Based on the UTAUT model and previous empirical studies, the following hypotheses are proposed:

**H1.**  *Effort expectancy (EE) has a positive effect on pre-service teachers’ behavioral intention (BI) to employ GenAI in teaching*.

**H2.**  *Facilitating conditions (FC) can positively influence pre-service teachers’ behavioral intention (BI) to employ GenAI in teaching.*

**H3.**  *Social influence (SI) can positively influence pre-service teachers’ behavioral intention (BI) to employ GenAI in teaching*.

**H4.**  *Performance expectancy (PE) has a positive effect on pre-service teachers’ behavioral intention (BI) to employ GenAI in teaching*.

#### 2.2.2. GenAI Anxiety

Anxiety, as conceptualized by Bandura [57], is a negative emotional response that negatively influences an individual’s intention to engage in specific tasks. In the context of technology adoption, this phenomenon is referred to as “technology anxiety”, which encompasses users’ concerns regarding their capability to effectively utilize technology-based tools [58]. It is frequently considered an external variable on the UTAUT model in numerous studies, as a meta-analysis revealed [59]. In the present study, GenAI anxiety is defined as a comprehensive emotional state that includes fear of using GenAI technology and concerns about its potential threats.

Interestingly, anxiety towards technology may have a dual effect on behavioral intention. Researchers have observed that anxiety can either facilitate or hinder learning and technology adoption [60]. On the one hand, the negative impact of anxiety on technology’s adoption has been widely acknowledged by researchers [38,61]. Studies conducted by Gunasinghe et al. [62], Huang [63], and Maican, Cazan, Lixandroiu, and Dovleac [49] indicated that anxiety can directly impede individuals’ intentions to employ new technologies. Furthermore, technology anxiety negatively affects teachers’ beliefs concerning specific technologies, particularly with regard to performance expectancy [64,65] and effort expectancy [61,66]. On the other hand, facilitating anxiety, as described by Piniel [67], elicits an approach behavior that positively influences motivated learning behavior. In this context, anxiety about GenAI technology may also encourage the efforts and persistence of pre-service teachers to enhance their GenAI usage skills. Furthermore, anxieties about GenAI potentially displacing certain jobs and occupations, which have also been noted in other research [68], might compel pre-service teachers to enhance their professional and technical competencies [60]. Consequently, it can be inferred that GenAI anxiety not only presents challenges but also acts as a catalyst for increasing pre-service teachers’ intention to engage with and master this emerging technology.

Based on the discussion above, this study proposes the following hypotheses:

**H5.**  *GenAI anxiety (ANX) impacts pre-service teachers’ effort expectancy (EE) regarding the adoption of GenAI in teaching*.

**H6.**  *GenAI anxiety (ANX) impacts pre-service teachers’ performance expectancy (PE) regarding the adoption of GenAI in teaching*.

**H7.**  *GenAI anxiety (ANX) impacts pre-service teachers’ behavioral intention (BI) regarding the adoption of GenAI in teaching*.

#### 2.2.3. GenAI TPACK

As a highly influential and fundamental framework in the realm of educational technology [69], technological pedagogical content knowledge (TPACK) is extensively employed to depict the expertise of educators in effectively incorporating technology into their instructional methodologies [70]. The concept of TPACK initially originated from Shulman’s notion of pedagogical and content knowledge (PCK) [71], which was subsequently expanded by Mishra and Koehler [72] to include technology. TPACK encompasses the integration of technological, pedagogical, and content knowledge, giving rise to four distinct types of knowledge: technological content knowledge (TCK), pedagogical content knowledge (PCK), technological pedagogical knowledge (TPK), and technology pedagogical content knowledge (TPACK) [73]. In previous studies on technology acceptance, TPACK has been frequently considered a valuable complement to the UTAUT model [37,74]. Some scholars even suggested its incorporation into the model for better explanations and conceptualizations of teaching practices that utilize technology [75,76].

Empirical studies, such as those conducted by Bardakcı and Alkan [74] and Lai Wah and Hashim [77], have suggested that TPACK has a significant impact on teachers’ intentions toward technology adoption. However, the integration of TPACK within the UTAUT model has received relatively less attention in existing research. To address this gap, recent studies, like those conducted by Yang et al. [78], have shed light on this area. Their research on K–12 teachers found that TPACK positively impacted technology acceptance, particularly influencing perceptions related to usefulness and ease of use, which are similar to performance expectancy and effort expectancy in the UTAUT model. Furthermore, research by An, Chai, Li, Zhou, Shen, Zheng, and Chen [37] further supported the notion that TPACK has a positive impact on both effort expectancy and performance expectancy among K–12 English teachers. Notably, according to a study that investigates the factors affecting EFL teachers’ adoption of Web 2.0 technologies, TPACK had the most significant effects on performance expectancy and effort expectancy compared to other factors considered [76]. Based on these research results, the following hypotheses are addressed:

**H8.**  *GenAI technological pedagogical and content knowledge (GenAI TPACK) has a positive effect on pre-service teachers’ effort expectancy (EE).*

**H9.**  *GenAI technological pedagogical and content knowledge (GenAI TPACK) has a positive effect on pre-service teachers’ performance expectancy (PE).*

#### 2.2.4. Technology Self-Efficacy

Self-efficacy refers to individuals’ confidence in their ability to proficiently organize and execute the necessary actions required to achieve a specific level of performance [79]. In this research, technology self-efficacy is defined as pre-service teachers’ confidence in their capacity to proficiently utilize GenAI technology [51].

Self-efficacy involves the regulation of cognitive, emotional, and behavioral skills that are crucial for effective task performance (Yeşilyurt et al., 2016). In other words, technology self-efficacy significantly shapes individuals’ perceptions of technology and governs their emotional responses to it. Specifically, it affects individuals’ evaluation of their own capabilities and perceived difficulty in utilizing technologies [80], influences their motivations, focuses, and efforts, as well as feelings of anxiety or self-doubt [81,82]. Research also indicates that technology self-efficacy is a key predictor of effort expectancy or perceived ease of use of technology [43,83]. Moreover, it has also been proven that technology self-efficacy, or an individual’s confidence in their ability to effectively utilize specific technologies, has a substantial impact on reducing anxiety towards technology [84].

According to Bandura et al. [85], possessing a high level of self-efficacy is essential for acquiring skills and knowledge, as well as maintaining task focus. Researchers have also emphasized that competence is built upon confidence, particularly in terms of technology self-efficacy, since the acquisition of knowledge and skills relies on gradual improvements and successful repetition, forming a “confidence/competence loop” [86]. Consequently, individuals with higher levels of technology self-efficacy tend to exert more effort and engagement [87], which improves their skills and competence when using technology. This enhanced proficiency will further influence their perception of the usefulness of technology. In other words, technology self-efficacy positively impacts teachers’ performance expectancy, as evidenced by various studies [88,89,90].

In addition, teachers with higher technology self-efficacy are more open to adopting innovative educational concepts and are inclined to explore diverse teaching methods that incorporate new technologies, thereby providing students with a wide range of unique learning opportunities [91,92]. Technology self-efficacy is thus considered to be “a necessary condition for technology integration” [93]. Previous studies have also confirmed the pivotal role of teachers’ technology self-efficacy in their TPACK [94,95]. Based on these findings, the following hypotheses are formulated:

**H10.**  *Pre-service teachers’ technology self-efficacy (TSE) negatively influences their GenAI anxiety (ANX).*

**H11.**  *Pre-service teachers’ technology self-efficacy (TSE) has a positive effect on their GenAI TPACK (TPACK).*

**H12.**  *Pre-service teachers’ technology self-efficacy (TSE) has a positive effect on their effort expectancy (EE).*

**H13.**  *Pre-service teachers’ technology self-efficacy (TSE) has a positive effect on their performance expectancy (PE).*

**Figure 1:**
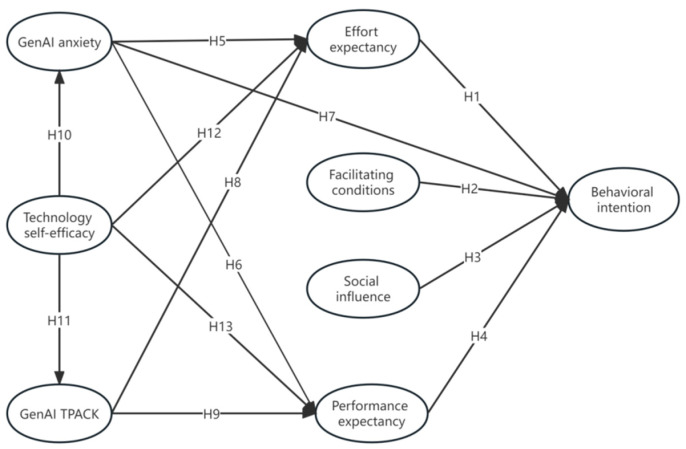
The hypothesized research model.

The hypothesis model illustrating the relationships mentioned above is presented in Figure 1.

## 3. Methods

### 3.1. Context and Participants

Towards the end of 2022, the launch of ChatGPT, a GenAI system, sparked extensive debates across various industries, including education. Numerous high-tech enterprises in China then expedited the development of GenAI tools. For instance, XIVO Whiteboard facilitates intelligent lesson preparation for teachers while enabling classroom feedback and learning analysis; Squirrel AI offers students personalized learning paths and precise teaching programs; and KU Xunfei’s intelligent education platform encompasses features like intelligent writing assessment, automated speaking evaluation, and personalized learning recommendations, among others. These advanced GenAI tools integrate state-of-the-art technologies such as natural language processing and deep learning to offer intelligent assistance and support in the field of education. Due to their extensive impact and recognition among educators in both formal (K–12) and informal educational settings in China, these tools were selected as representatives of generative AI to investigate pre-service teachers’ willingness to adopt them.

This study utilized data gathered via an anonymous online survey administered from August to October 2023. It was explicitly mentioned that participation was entirely voluntary. After excluding 60 outliers and repeated questionnaires, a total of 606 valid samples were collected, of which 473 (78.1%) were women. The educational level of the participants was varied: 61.6% were undergraduate students, while 40.4% were engaged in postgraduate studies. These participants represented a broad range of academic disciplines, with a significant portion having previously engaged in technical courses and obtained relevant training experience. The sample profiles of the participants are summarized in Table 1.

### 3.2. Instruments

A multi-item survey was employed to explore the willingness of pre-service teachers to integrate GenAI into their teaching. This survey consisted of two sections, with the initial part gathering demographic data from the respondents. The second section consisted of 41 items designed to assess performance expectancy, effort expectancy, social influence, facilitating conditions, TPACK, technology self-efficacy, GenAI anxiety, and behavioral intention. A 5-point Likert scale, ranging from 1 (strongly disagree) to 5 (strongly agree), was utilized to score all items. Recognizing potential language barriers faced by some pre-service teachers, the questionnaire was rendered in Chinese. To maintain the precision and fidelity of the questionnaire in its Chinese format, this process adhered to the established standards of translation and back-translation as outlined by Brislin [96].

#### 3.2.1. Technology Self-Efficacy

This study used Dong et al.’s Technology Self-Efficacy Scale [97] to measure pre-service teachers’ self-efficacy in using GenAI technologies. In order to fit the context of this study, the word “technology” was replaced with “GenAI technology or tool” in all items. It contains four questions, and sample questions include “I can always manage to solve difficult problems using GenAI tools if I try hard enough”. This scale showed good reliability, with a Cronbach’s coefficient of 0.857.

#### 3.2.2. Gen Anxiety

The AI Anxiety Scale, developed by Wang and Wang [60], was used in this study to measure pre-service teachers’ anxiety levels when using GenAI tools. The initial item, “artificial intelligence”, was changed to “GenAI technology/products” in order to better align with the study’s focus. The scale consists of four dimensions: learning anxiety (five items, e.g., “Learning to understand all of the special functions associated with GenAI technology/products makes me anxious”), Job replacement anxiety (three items, e.g., “I am afraid that a GenAI technology/products may make us dependent”), sociotechnical blindness (three items, e.g., “I am afraid that a GenAI technology/products may be misused”), and AI configuration anxiety (three items, e.g., “I find humanoid GenAI robots scary”). With Cronbach’s coefficients of 0.886, 0.886, 0.887, and 0.924 for each of the four dimensions and an overall Cronbach’s coefficient of 0.908, all dimensions exhibited strong reliability.

#### 3.2.3. TPACK

The AI-TPACK Scale [98] was used to measure pre-service teachers’ knowledge and skill levels regarding the integration of GenAI techniques into their teaching practice. The original items were modified to align with the characteristics of the pre-service teachers involved in this study and the specific circumstances of GenAI. The scale comprises four items, with the sample item being “I possess the knowledge and ability to instruct a subject using GAI-based tools while employing various teaching strategies”. The Cronbach’s coefficient in this study was 0.908.

#### 3.2.4. UTAUT

The scale developed by Venkatesh, Morris, Davis, and Davis [38] was used to measure users’ views of performance expectancy, effort expectancy, facilitating conditions, social influence, and behavioral intentions regarding technologies. This study used its adapted scale developed by An, Chai, Li, Zhou, Shen, Zheng, and Chen [37], which fits better with the context of using GenAI in the educational context of this study. The scale consists of five subscales: (1) performance expectancy, consisting of four items (e.g., “GenAI can help me improve the quality of teaching”); and (2) effort expectancy, which consists of four items (e.g., “GenAI teaching systems are easy to operate for me”); (3) facilitating conditions, encompassing four items (e.g., “When I need to use GenAI in teaching, my school will provide help for me”); (4) social influence, containing three items (e.g., “Teachers around me who are good at using GenAI will have more respect”); and (5) behavioral intention, consisting of four items (e.g., “I intend to use GenAI in teaching in the future”). All of these constructs showed satisfactory reliability, and their Cronbach’s coefficients were 0.943, 0.906, 0.917, 0.873, and 0.892, respectively.

### 3.3. Data Analysis

In this study, the data were analyzed in the following manner: Initially, we used SPSS 26.0 for descriptive statistical analysis. Following this, Confirmatory Factor Analysis (CFA) and second-order CFAs were conducted. The purpose of the second-order CFA was to validate the measurement for the structures related to GenAI anxiety, which contains four sub-dimensions: anxiety regarding learning, job replacement, sociotechnical blindness, and AI configuration. Subsequently, latent variable path analysis was conducted to assess the hypotheses formulated in the study.

## 4. Results

### 4.1. Descriptive Statistics

Table 2 presents the descriptive statistics of all constructs. The mean values of these constructs varied between 2.53 and 3.84. In terms of distribution characteristics, these observed values for skewness and kurtosis were within the generally accepted thresholds, which are |1| and |2|, respectively, suggesting that the distribution of the data approximated a normal distribution [99].

### 4.2. Examination of the Measurement Model

The measurement model was assessed comprehensively, including the assessment of internal consistency reliability, convergent validity, and discriminant validity. The reliability and validity of all constructs were found to be satisfactory, as demonstrated in Table 3. The internal consistency of constructs was examined through Cronbach’s alpha coefficients, surpassing the benchmark of 0.70 [100]. The construct’s reliability was assessed using composite reliability (CR). A CR value above 0.70 indicates good reliability [101]. The results showed that all CR values were higher than 0.80. Convergent validity was evaluated using average variance extracted (AVE), and all AVE values exceeded the recommended threshold of 0.50 [101]. Meanwhile, the factor loading, which ranged from 0.624 to 0.936, exceeded the recommended minimum of 0.50 [102]. The measurement model also demonstrated satisfactory fit indices: χ^2^/df = 2.15 (<5.0), RMSEA = 0.044 (<0.08), SRMR = 0.038 (<0.08), CFI = 0.958 (>0.90), and TLI = 0.952 (>0.90) [103].

As suggested by Hair, Black, Babin, and Anderson [103], the scale’s discriminant validity was measured by comparing the square roots of the AVE for each construct with the correlations among these constructs. This comparison, detailed in Table 4, showed that all square root values of the AVEs were higher than the correlations between the constructs, indicating strong discriminant validity.

### 4.3. Examination of the Hypothesized Model

**Figure 2:**
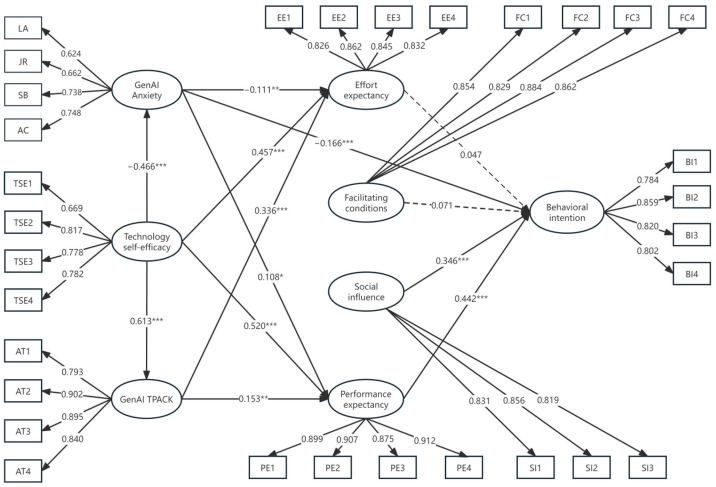
Results for the structural model. Note: * *p* < 0.05, ** *p* < 0.01, *** *p* < 0.001.

The structural equation model (SEM) exhibited favorable fit indices: χ^2^/df = 2.636 (<5.0), CFI = 0.937 (>0.90), TLI = 0.932 (>0.90), RMSEA = 0.052 (<0.08), and SRMR = 0.077 (<0.08). The empirical validation of the research model, as depicted in Figure 2, revealed that 11 out of the 13 proposed hypotheses received support (refer to Table 5 for details).

Among BI-related hypotheses, those with effort expectancy and facilitating conditions were not significant, while those established with social influence, performance expectancy, and GenAI anxiety were significant. Hence, H1 and H2 were not supported, while H3, H4, and H7 were supported. In terms of GenAI anxiety, GenAI anxiety had a negatively significant predictive effect on effort expectancy and performance expectancy, and therefore H5 and H6 were supported. As for the relationship between GenAI TPACK and UTAUT for GenAI, the hypothesized paths of GenAI TPACK to effort expectancy and performance expectancy were significant, so H8 and H9 were supported. In addition to these, technology self-efficacy had a significant negative effect on GenAI anxiety while having a significant positive effect on GenAI TPACK, effort expectancy, and performance expectancy, and thus, all of the hypotheses proposed in the technology self-efficacy context (H10–H13) were supported. In addition, technology self-efficacy with GenAI TPACK was the strongest relationship in the model.

## 5. Discussion

The objective of the research was to investigate the determinants and mechanisms influencing pre-service teachers’ intentions to integrate GenAI technology into their teaching practices. A hypothetical model was developed to investigate the internal and external factors that affect their intentions. Out of all the hypotheses, a total of 11 were supported, while 2 were not. Therefore, this hypothetical model was generally validated. The findings are further discussed below.

### 5.1. The UTAUT Model

The UTAUT model posits that an individual’s inclination to utilize technology is primarily impacted by four fundamental elements: performance expectancy, effort expectancy, social influence, and facilitating conditions [38]. However, this study found that only two of these factors—performance expectancy and social influence—significantly impacted pre-service teachers’ behavioral intention toward using GenAI technology in their teaching practices. This suggests that regardless of the complexity of GenAI teaching systems or schools’ ability to provide resources and support, there is a limited impact on pre-service teachers’ willingness to adopt them.

The findings of this study validate that performance expectancy and social influence significantly impact pre-service teachers’ behavioral intention to incorporate GenAI into teaching practices, which aligns with previous research [104,105,106]. Researchers have observed that, in a highly collectivist culture, Chinese teachers are particularly influenced by important social connections [107]. The recommendations from their peers and teachers significantly shape these pre-service teachers’ behavioral intentions toward embracing GenAI. Meanwhile, in terms of performance expectancy, it was the most influential predictor of pre-service teachers’ intentions among all factors considered according to the quantitative results. This significant finding is also consistent with previous research on English teachers’ behavioral intention to use AI in middle schools [37].

The results of this study suggested that preservice teachers’ effort expectancy and facilitating conditions did not influence their behavioral intentions. Notably, previous research employing UTAUT to examine various technologies suggested that its key factors might have differential effects on technology acceptance behavior. For example, a study conducted by Hu, Laxman, and Lee [106] explored the acceptance of mobile technology among academics in Chinese higher education and demonstrated that effort expectancy did not exert any significant impact on intentions to adopt mobile technology, while facilitating conditions significantly influenced their intentions. However, the findings of a study investigating the factors influencing pre-service teachers’ intentions to utilize a learning management system revealed that effort expectancy significantly influenced attitudes towards usage, while facilitating conditions did not exhibit any impact on attitudes [55]. These divergent results may be attributed to variations in user group characteristics, the technology under investigation, and the cultural context [108].

In this study, the insignificant effect of effort expectancy on behavioral intention may be attributed to the applicability of UTAUT to different user groups. Most survey respondents were pre-service teachers, predominantly engaged in higher education, with 76.7% having technical training. Compared to other types of users, higher-education academics, as professionals, may have a relatively high level of competence and adaptability to novel technologies [109]. In addition, as pre-service teachers, they may value its educational benefits more than the simplicity of operation. Researchers pointed out that, despite potential operational challenges, users’ willingness to adopt technologies is rooted in the effectiveness and accomplishments they bring [110].

Our study revealed that the influence of facilitating conditions on pre-service teachers’ behavioral intention to utilize GenAI was not statistically significant, potentially due to the user-friendly technical features and convenient external support environment offered by GenAI. The ease of use of GenAI applications may diminish the impact of facilitating conditions on pre-service teachers’ inclination [111]. Meanwhile, facilitated by rich online resources related to GenAI technology, pre-service teachers are able to cope with difficulties they meet through a self-sourced learning approach. Accordingly, the impact of facilitating conditions on their inclination to incorporate GenAI into future teaching practices may not be crucial.

### 5.2. GenAI Anxiety

Previous studies indicate that anxiety about technologies can negatively impact users’ willingness to use them. Aligning with research by Ni and Cheung [110], Almisad and Alsalim [112], and Kamalasena and Sirisena [113], GenAI anxiety’s clear negative effect existed on factors like effort expectancy, performance expectancy, and behavioral intention. Specifically, when pre-service teachers harbor concerns about GenAI, they tend to perceive its use as more challenging and less beneficial, thus reducing their inclination to incorporate it into future teaching practices.

As pointed out by Holzmann et al. [114], advancements in technology often bring complexity and uncertainty. As an emerging technology, GenAI also presents various challenges, such as potential job replacement, privacy and transparency concerns, algorithmic biases, widening socio-economic disparities, and unethical utilization of technology [115]. Such challenges are likely to evoke anxiety among pre-service teachers, thereby influencing their perception of effort expectancy, performance expectancy, and behavioral intention toward the adoption of GenAI in teaching. In fact, anxieties towards technology, such as privacy and over-reliance on technology, are frequently cited as significant barriers to adopting new technological tools in previous studies [68,107,116].

### 5.3. GenAI TPACK

The results indicated that GenAI TPACK positively impacted pre-service teachers’ effort expectancy and performance expectancy. In other words, those who are skilled in integrating GenAI into their pedagogical and subject-teaching approaches tend to view GenAI as an effective and user-friendly tool for improving the quality and efficiency of teaching practices. This finding reinforces previous research that TPACK influences teachers’ perceptions of effort expectancy (or perceived ease of use) and performance expectancy (or perceived usefulness) [117,118,119].

Pre-service teachers with a higher level of TPACK have a deeper understanding of how to effectively integrate GenAI technology into teaching and learning activities, including the utilization of GenAI for developing instructional materials, designing curriculum, offering personalized tutoring, and facilitating assessment [5]. Joo, Park, and Lim’s [119] research also suggested that a strong TPACK foundation enables pre-service teachers to better comprehend how technology improves pedagogical performance and to effectively foster their confidence in utilizing technology. Their better grasp and higher level of confidence in integrating GenAI technology into teaching make them believe that it can improve teaching outcomes and serve as a user-friendly tool rather than a burden for teachers, thereby influencing their performance expectancy and effort expectancy.

### 5.4. Technology Self-Efficacy

The results of this study proved the negative impact of technology self-efficacy on GenAI anxiety and its positive influences on effort expectancy, performance expectancy, and GenAI TPACK. Consistent with prior research findings [90,120,121], individuals with higher levels of self-efficacy are more inclined to perceive technology as a beneficial and easy-to-use tool for teaching practices. According to Rahmawati [122], self-efficacy stimulates a greater inclination towards engaging in a task, empowering individuals to exert maximum effort in order to successfully complete the task. Therefore, those possessing high technology self-efficacy are more receptive to new GenAI apps and exhibit a stronger willingness to explore their complex functions deeply. Consequently, their perception of complexity diminished while recognizing the practical advantages of integrating technology into education.

This study also revealed that technology self-efficacy had a significant impact on pre-service teachers’ GenAI TPACK. These findings reinforce prior research conclusions regarding the significant influence of teachers’ technology self-efficacy on their TPACK [95,123,124]. Researchers have demonstrated that teachers’ self-efficacy beliefs serve as valuable indicators for successful technology integration [93,125], which is central to TPACK.

In line with previous research [126,127,128], the findings of this study verified technology self-efficacy’s positive effect on GenAI anxiety. Higher levels of technology self-efficacy were found to be effective in reducing GenAI anxiety, while lower levels of technology self-efficacy were associated with increased anxiety. Previous studies have also suggested that ignorance often leads to anxiety [129], which indicates that a lack of controllability and knowledge about technology may increase anxiety [130]. The concept of controllability aligns closely with technology self-efficacy since both reflect an individual’s belief in their competence to manage technology [131]. Therefore, pre-service teachers with strong technology self-efficacy tend to be more confident in their skills and knowledge related to the usage and management of GenAI, and this sense of mastery over GenAI tools can effectively reduce the likelihood of feeling overwhelmed or anxious when adopting GenAI.

## 6. Implications

This study’s findings are instructive for both theoretical frameworks and pedagogical practices. Theoretically, the findings expand on the UTAUT model and clarify the intricate relationships between various predictors of pre-service teachers’ behavioral intention to use GenAI in teaching. Although the UTAUT model has been used to study a variety of technology behavioral intentions in different contexts, it is essentially a broad technology adoption model rather than context-specific [132]. The four key variables in it represent technological factors (performance expectancy and effort expectancy) and environmental factors (facilitating conditions and social influence), while ignoring the individual characteristics of users [133]. This study focuses on the effectiveness of the UTAUT model in a GenAI context while emphasizing the personal characteristics of the pre-service teachers. The results suggested that pre-service teachers’ effort expectancy and facilitating conditions for GenAI technologies did not influence their behavioral intention and highlighted the crucial role of psychological factors (such as self-efficacy and anxiety) and knowledge (TPACK) in shaping perspectives towards GenAI. These findings offer insights into how the UTAUT model can be adjusted and improved in order to better assess the predictors of technology acceptance, particularly in the context of GenAI.

In practice, this research also offers evidence and guidance for the design and implementation of teacher training programs using GenAI technologies. The results indicated that performance expectancy and social influence had a significant impact on the intentions of pre-service teachers to utilize GenAI technology. Therefore, institutions of higher education, particularly in teacher education programs, should incorporate courses or establish continuous professional development initiatives focused on GenAI [134]. These training programs ought to clearly demonstrate the pedagogical objectives and outcomes achievable through GenAI technology. Meanwhile, showcasing successful GenAI-assisted teaching cases may enable pre-service teachers to visualize the advantages of this technology. Additionally, it is crucial to invite experienced experts in the field of GenAI teaching to provide guidance and share their knowledge while encouraging pre-service teachers to form a learning community amongst themselves in order to enhance their identification with GenAI.

According to the findings, anxiety about GenAI significantly reduced pre-service teachers’ intentions to use it. To address anxieties related to GenAI, such as unfamiliarity, concerns about privacy, and ethical issues, a collaborative effort involving multiple stakeholders, including policymakers, educators, and technology developers, should be undertaken [68]. The formulation of relevant policies is essential for monitoring, warning, and governing the potential risks associated with GenAI. Additionally, teacher educators should incorporate content on technical operations and teaching practices related to GenAI technology in order to alleviate pre-service teachers’ anxiety stemming from unfamiliarity. Furthermore, technology developers should actively promote the iteration and upgrading of GenAI technologies as a means to address potential risks. Consequently, the benefits of GenAI in education can be maximized while associated risks can be minimized, thus ensuring that its integration into teaching and learning aligns with competencies, privacy standards, and ethical principles.

In addition, technology self-efficacy and TPACK level had a positive impact on pre-service teachers’ performance expectancy, which might affect their intentions to use technology. Teachers’ innovativeness and TPACK can be improved by offering them opportunities for designing curriculum materials assisted by GenAI technologies [135]. Furthermore, building a supportive technological community, which can be achieved through providing positive role models and peer support, can successfully encourage collaboration and information sharing among teachers [136] and, therefore, enhance per-service teachers’ social influence and self-efficacy in designing GenAI-assisted teaching.

## 7. Limitations and Suggestions

Given the constraints of limited time and scope, this study still has some limitations that could be addressed in future research. Firstly, the study’s participant pool was limited to pre-service teachers from China. The acceptance of technology may vary in different cultural and educational contexts. For instance, in a study examining the acceptance of m-learning technologies among university students in Saudi Arabia, the presence of facilitating conditions did not influence their willingness to use them [133]. However, a study conducted on Indonesian university students’ willingness to adopt an m-learning system revealed that all four key factors of UTAUT positively influenced their intention to use it [137]. This implies that the influence of technology can significantly differ across various contexts and individuals’ characteristics. The present study offers only subtle insights into how pre-service teachers perceive GenAI within a specific context. Future research could employ a multi-group comparative analysis involving educators from diverse cultural or educational backgrounds, thereby revealing more nuanced insights and validating the generalizability of the findings. Meanwhile, there are limitations inherent in the survey design and reliance on self-reported data. The structured nature of the questionnaire may not fully capture the contextual nuances of participants’ experiences or behaviors, potentially limiting its comprehensiveness. Additionally, relying solely on self-reporting introduces potential methodological biases. Future research could consider incorporating multiple data sources to enhance cross-validation and ensure result validity. Additionally, the study’s reliance on cross-sectional data may limit the understanding of the progression of pre-service teachers’ perceptions over time. To acquire a more extensive comprehension of how these perceptions evolve and the implementation process of GenAI in education, adopting a longitudinal approach would be beneficial to offer insights into their dynamic transition. Additionally, this research did not encompass the examination of moderating factors, like demographic elements such as age, gender, and experience. According to Bower, Torrington, Lai, Petocz, and Alfano [36], the behavioral intention of utilizing technologies may vary based on factors such as teaching experience, teaching level, subject area, region, and gender. Future studies are encouraged to delve into these aspects and investigate how these demographic variables may moderate the relationships between predictors and individuals’ behavioral intentions to use GenAI technology, thus helping tailor more inclusive and effective educational technologies and strategies.

## 8. Conclusions

Grounded in the UTAUT model, this study provides a comprehensive analysis of the determinants influencing pre-service teachers’ behavioral intention to include GenAI in their teaching practices. Key findings indicated that social influence, performance expectancy, and GenAI anxiety were substantial determinants of their behavioral intention. Notably, variables such as effort expectancy and facilitating conditions did not exert significant influences on behavioral intention. In addition, this research highlighted the pivotal role of technology self-efficacy, GenAI anxiety, and TPACK in shaping pre-service teachers’ effort expectancy and performance expectancy in GenAI. Overall, this study offers valuable understanding regarding the complex interplay among various factors affecting pre-service teachers’ perspectives and intentions toward GenAI technology. These findings offer a detailed blueprint for educators and policymakers with a theoretical foundation and empirical validation to encourage pre-service teachers to adopt generative artificial intelligence in their teaching practices.

## Figures and Tables

**Table 1:** Demographic data of participants (N = 606).

Profile	Category	Frequency	Percentage (%)
Gender	Male	133	21.9
	Female	473	78.1
Age	≤23	422	69.6
	24–29	162	26.7
	30–35	15	2.5
	≥36	7	1.2
Level of degree	Undergraduate	373	61.6
	Postgraduate	233	38.4
Discipline background	Pedagogy	204	33.7
	Science and Engineering	80	13.2
	Liberal arts	205	33.8
	Arts and PE	47	7.8
	Other	70	11.6
Prior relevant training experience	Yes	465	76.7
	No	141	23.3
Familiarity level of GenAI	Completely Unfamiliar	51	8.4
	Somewhat Familiar	470	77.6
	Fairly Familiar	67	11.1
	Very Familiar	18	3
Frequency of using GenAI apps	Never	302	49.8
	Occasionally	240	39.6
	Frequently	42	6.9
	Regularly	22	3.6

**Table 2:** Descriptive statistics (N = 606).

Construct	Mean	SD	Skewness	Kurtosis
Technology self-efficacy	3.58	0.72	−0.56	1.36
Learning	2.53	0.78	0.21	−0.54
Job replacement	3.28	0.92	−0.40	−0.41
Sociotechnical blindness	3.44	0.91	−0.61	−0.04
AI configuration	2.63	1.01	0.12	−0.85
GenAI Anxiety	2.91	0.68	−0.16	−0.20
GenAI TPACK	3.35	0.78	−0.48	0.07
Performance expectancy	3.83	0.63	−0.63	1.09
Effort expectancy	3.38	0.74	−0.36	0.34
Facilitating conditions	3.43	0.77	−0.76	1.10
Social influence	3.51	0.71	−0.48	1.08
Behavioral intention	3.84	0.60	−0.28	0.45

**Table 3:** Results of the measurement model.

Construct	Item	Loadings	CR	AVE	α
Technology self-efficacy			0.848	0.583	0.857
	TSE1	0.669			
	TSE2	0.817			
	TSE3	0.778			
	TSE4	0.782			
GenAI Anxiety			0.845	0.525	0.908
Learning		0.624	0.888	0.614	0.886
	LA1	0.735			
	LA2	0.759			
	LA3	0.815			
	LA4	0.844			
	LA5	0.759			
Job replacement		0.662	0.893	0.737	0.886
	JR1	0.902			
	JR2	0.903			
	JR3	0.762			
Sociotechnical blindness		0.738	0.892	0.734	0.887
	SB1	0.861			
	SB2	0.916			
	SB3	0.789			
AI configuration		0.748	0.926	0.806	0.924
	AC1	0.936			
	AC2	0.926			
	AC3	0.828			
GenAI TPACK			0.918	0.737	0.908
	AT1	0.793			
	AT2	0.902			
	AT3	0.895			
	AT4	0.840			
Performance expectancy			0.944	0.807	0.943
	PE1	0.899			
	PE2	0.907			
	PE3	0.875			
	PE4	0.912			
Effort expectancy			0.906	0.708	0.906
	EE1	0.826			
	EE2	0.862			
	EE3	0.845			
	EE4	0.832			
Facilitating conditions			0.917	0.735	0.917
	FC1	0.854			
	FC2	0.829			
	FC3	0.884			
	FC4	0.862			
Social influence			0.874	0.698	0.873
	SI1	0.831			
	SI2	0.856			
	SI3	0.819			
Behavioral intention			0.889	0.667	0.892
	BI1	0.784			
	BI2	0.859			
	BI3	0.820			
	BI4	0.802			

**Table 4:** Construct correlations and discriminant validity.

Construct	TSE	ANX	TPACK	PE	EE	FC	SI	BI
TSE	0.764							
ANX	−0.466	0.725						
TPACK	0.613	−0.286	0.858					
PE	0.664	−0.394	0.503	0.898				
EE	0.715	−0.420	0.648	0.516	0.841			
FC	0.693	−0.323	0.425	0.460	0.495	0.857		
SI	0.657	−0.307	0.403	0.436	0.470	0.673	0.835	
BI	0.682	−0.489	0.470	0.716	0.543	0.585	0.660	0.817

Note: Each construct’s square root values of AVE are represented by the diagonal entries in the table. The non-diagonal values indicate the correlation coefficients for each factor. All correlations were found to be statistically significant (*p* < 0.001). ANX = GenAI anxiety; TSE = Technology self-efficacy; TPACK = GenAI technological pedagogical content knowledge; EE = Effort expectancy; FC = Facilitating conditions; SI = Social influence; PE = Performance expectancy; BI = Behavioral intention.

**Table 5:** Hypothesis testing results.

Hypothesis	Path	Std β	Std Error	*p*-Value	Conclusion
H1	EE→BI	0.047	0.045	0.298	Not Supported
H2	FC→BI	0.071	0.048	0.136	Not Supported
H3	SI→BI	0.346	0.047	0.000	Supported
H4	PE→BI	0.442	0.039	0.000	Supported
H5	ANX→EE	−0.111	0.041	0.007	Supported
H6	ANX→PE	−0.108	0.045	0.017	Supported
H7	ANX→BI	−0.166	0.039	0.000	Supported
H8	TPACK→EE	0.336	0.042	0.000	Supported
H9	TPACK→PE	0.153	0.046	0.001	Supported
H10	TSE→ANX	−0.466	0.043	0.000	Supported
H11	TSE→TPACK	0.613	0.030	0.000	Supported
H12	TSE→EE	0.457	0.047	0.000	Supported
H13	TSE→PE	0.520	0.050	0.000	Supported

Note: ANX = GenAI anxiety; TSE = Technology self-efficacy; TPACK = GenAI technological pedagogical content knowledge; EE = Effort expectancy; FC = Facilitating conditions; SI = Social influence; PE = Performance expectancy; BI = Behavioral intention.

## Data Availability

The datasets used in the current study are available from the corresponding author upon reasonable request.

## References

[1] Thayyib P., Mamilla R., Khan M., Fatima H., Asim M., Anwar I., Shamsudheen M., Khan M.A. (2023). State-of-the-Art of Artificial Intelligence and Big Data Analytics Reviews in Five Different Domains: A Bibliometric Summary. Sustainability.

[2] Peres R., Schreier M., Schweidel D., Sorescu A. (2023). On ChatGPT and beyond: How generative artificial intelligence may affect research, teaching, and practice. Int. J. Res. Mark..

[3] García-Peñalvo F.J., Llorens Largo F., Vidal J. (2024). The new reality of education in the face of advances in generative artificial intelligence. Rev. Iberoam. De Educ. A Distancia.

[4] Lim W.M., Gunasekara A., Pallant J.L., Pallant J.I., Pechenkina E. (2023). Generative AI and the future of education: Ragnarök or reformation? A paradoxical perspective from management educators. Int. J. Manag. Educ..

[5] Baidoo-Anu D., Ansah L.O. (2023). Education in the era of generative artificial intelligence (AI): Understanding the potential benefits of ChatGPT in promoting teaching and learning. J. AI.

[6] Pratama M.P., Sampelolo R., Lura H. (2023). Revolutionizing education: Harnessing the power of artificial intelligence for personalized learning. Klasikal J. Educ. Lang. Teach. Sci..

[7] Chassignol M., Khoroshavin A., Klimova A., Bilyatdinova A. (2018). Artificial Intelligence trends in education: A narrative overview. Procedia Comput. Sci..

[8] Herft A. (2023). A Teacher’s Prompt Guide to ChatGPT Aligned with ‘What Works Best’. https://bit.ly/3K9z6my.

[9] Kikalishvili S. (2023). Unlocking the potential of GPT-3 in education: Opportunities, limitations, and recommendations for effective integration. Interact. Learn. Environ..

[10] Chen L., Chen P., Lin Z. (2020). Artificial Intelligence in Education: A Review. IEEE Access.

[11] Mercader C., Gairín J. (2020). University teachers’ perception of barriers to the use of digital technologies: The importance of the academic discipline. Int. J. Educ. Technol. High. Educ..

[12] Lassoued Z., Alhendawi M., Bashitialshaaer R. (2020). An exploratory study of the obstacles for achieving quality in distance learning during the COVID-19 pandemic. Educ. Sci..

[13] Kaplan-Rakowski R., Grotewold K., Hartwick P., Papin K. (2023). Generative AI and teachers’ perspectives on its implementation in education. J. Interact. Learn. Res..

[14] Joseph G.V., Thomas K.A., Nero A. (2021). Impact of technology readiness and techno stress on teacher engagement in higher secondary schools. Digit. Educ. Rev..

[15] Ishak N., Din R., Othman N. (2022). Teachers’ Perceptions and Challenges to the Use of Technology in Teaching and Learning during COVID-19 in Malaysia. Int. J. Learn. Teach. Educ. Res..

[16] Herodotou C., Rienties B., Boroowa A., Zdrahal Z., Hlosta M. (2019). A large-scale implementation of predictive learning analytics in higher education: The teachers’ role and perspective. Educ. Technol. Res. Dev..

[17] Kafyulilo A., Fisser P., Voogt J. (2016). Factors affecting teachers’ continuation of technology use in teaching. Educ. Inf. Technol..

[18] Peng R., Abdul Razak R., Hajar Halili S. (2023). Factors influencing in-service teachers’ technology integration model: Innovative strategies for educational technology. PLoS ONE.

[19] Teo T. (2011). Factors influencing teachers’ intention to use technology: Model development and test. Comput. Educ..

[20] Jin L., Xu Y., Deifell E., Angus K. (2021). Emergency remote language teaching and US-based college-level world language educators’ intention to adopt online teaching in postpandemic times. Mod. Lang. J..

[21] Celik I., Dindar M., Muukkonen H., Järvelä S. (2022). The promises and challenges of artificial intelligence for teachers: A systematic review of research. TechTrends.

[22] Chiu T.K., Chai C.-s. (2020). Sustainable curriculum planning for artificial intelligence education: A self-determination theory perspective. Sustainability.

[23] Wong J.Y., Oh P. (2023). Teaching physical education abroad: Perspectives from host cooperating teachers, local students and Australian pre-service teachers using the social exchange theory. Teach. Teach. Educ..

[24] Michalsky T. (2021). Integrating video analysis of teacher and student behaviors to promote Preservice teachers’ teaching meta-strategic knowledge. Metacognition Learn..

[25] Chao C.-M. (2019). Factors determining the behavioral intention to use mobile learning: An application and extension of the UTAUT model. Front. Psychol..

[26] Mohebi L. (2021). Theoretical models of integration of interactive learning technologies into teaching: A systematic literature review. Int. J. Learn. Teach. Educ. Res..

[27] Su J., Yang W. (2023). Unlocking the power of ChatGPT: A framework for applying generative AI in education. ECNU Rev. Educ..

[28] Luckin R., Cukurova M., Kent C., du Boulay B. (2022). Empowering educators to be AI-ready. Comput. Educ. Artif. Intell..

[29] Lameras P., Arnab S. (2021). Power to the Teachers: An Exploratory Review on Artificial Intelligence in Education. Information.

[30] Malik T., Dwivedi Y., Kshetri N., Hughes L., Slade E.L., Jeyaraj A., Kar A.K., Baabdullah A.M., Koohang A., Raghavan V. (2023). “So what if ChatGPT wrote it?” Multidisciplinary perspectives on opportunities, challenges and implications of generative conversational AI for research, practice and policy. Int. J. Inf. Manag..

[31] Xu W., Ouyang F. (2022). A systematic review of AI role in the educational system based on a proposed conceptual framework. Educ. Inf. Technol..

[32] Tlili A., Shehata B., Adarkwah M.A., Bozkurt A., Hickey D.T., Huang R., Agyemang B. (2023). What if the devil is my guardian angel: ChatGPT as a case study of using chatbots in education. Smart Learn. Environ..

[33] Cooper G. (2023). Examining Science Education in ChatGPT: An Exploratory Study of Generative Artificial Intelligence. J. Sci. Educ. Technol..

[34] Zawacki-Richter O., Marín V.I., Bond M., Gouverneur F. (2019). Systematic review of research on artificial intelligence applications in higher education–where are the educators?. Int. J. Educ. Technol. High. Educ..

[35] van den Berg G., du Plessis E. (2023). ChatGPT and generative AI: Possibilities for its contribution to lesson planning, critical thinking and openness in teacher education. Educ. Sci..

[36] Bower M., Torrington J., Lai J.W., Petocz P., Alfano M. (2024). How should we change teaching and assessment in response to increasingly powerful generative Artificial Intelligence? Outcomes of the ChatGPT teacher survey. Educ. Inf. Technol..

[37] An X., Chai C.S., Li Y., Zhou Y., Shen X., Zheng C., Chen M. (2023). Modeling English teachers’ behavioral intention to use artificial intelligence in middle schools. Educ. Inf. Technol..

[38] Venkatesh V., Morris M.G., Davis G.B., Davis F.D. (2003). User acceptance of information technology: Toward a unified view. MIS Q..

[39] Ning F., Yang Y., Zhu T., Bayarmaa T.-I., Ma N. Influence of Pre-service and In-service Teachers’ Gender and Experience on the Acceptance of AR Technology. Proceedings of the Foundations and Trends in Smart Learning: Proceedings of 2019 International Conference on Smart Learning Environments.

[40] Alotumi M. (2022). Factors influencing graduate students’ behavioral intention to use Google Classroom: Case study-mixed methods research. Educ. Inf. Technol..

[41] Oye N., Iahad N.A., Rahim N.A. (2014). The history of UTAUT model and its impact on ICT acceptance and usage by academicians. Educ. Inf. Technol..

[42] Agyei C., Razi Ö. (2022). The effect of extended UTAUT model on EFLs’ adaptation to flipped classroom. Educ. Inf. Technol..

[43] Islamoglu H., Kabakci Yurdakul I., Ursavas O.F. (2021). Pre-service teachers’ acceptance of mobile-technology-supported learning activities. Educ. Technol. Res. Dev..

[44] Wu C., Gong X., Luo L., Zhao Q., Hu S., Mou Y., Jing B. (2021). Applying control-value theory and unified theory of acceptance and use of technology to explore pre-service teachers’ academic emotions and learning satisfaction. Front. Psychol..

[45] Venkatesh V. (2000). Determinants of perceived ease of use: Integrating control, intrinsic motivation, and emotion into the technology acceptance model. Inf. Syst. Res..

[46] Ning Y., Dong C. Factors Influencing Pre-service Teachers’ Acceptance to Introduce Danmaku Video into Online Education. Proceedings of the 2021 International Symposium on Educational Technology (ISET).

[47] Maduku D.K. (2017). Understanding E-book continuance intention: Empirical evidence from E-book users in a developing country. Cyberpsychology Behav. Soc. Netw..

[48] Shen C.-W., Ho J.-T., Ly P.T.M., Kuo T.-C. (2019). Behavioural intentions of using virtual reality in learning: Perspectives of acceptance of information technology and learning style. Virtual Real..

[49] Maican C.I., Cazan A.-M., Lixandroiu R.C., Dovleac L. (2019). A study on academic staff personality and technology acceptance: The case of communication and collaboration applications. Comput. Educ..

[50] Teo T., Milutinović V., Zhou M. (2016). Modelling Serbian pre-service teachers’ attitudes towards computer use: A SEM and MIMIC approach. Comput. Educ..

[51] Gurer M.D. (2021). Examining technology acceptance of pre-service mathematics teachers in Turkey: A structural equation modeling approach. Educ. Inf. Technol..

[52] Fathi J., Ebadi S. (2020). Exploring EFL pre-service teachers’ adoption of technology in a CALL program: Obstacles, motivators, and maintenance. Educ. Inf. Technol..

[53] Kim J., Lee K.S.-S. (2022). Conceptual model to predict Filipino teachers’ adoption of ICT-based instruction in class: Using the UTAUT model. Asia Pac. J. Educ..

[54] Wong G.K. (2015). Understanding technology acceptance in pre-service teachers of primary mathematics in Hong Kong. Australas. J. Educ. Technol..

[55] Buabeng-Andoh C., Baah C. (2020). Pre-service teachers’ intention to use learning management system: An integration of UTAUT and TAM. Interact. Technol. Smart Educ..

[56] Yildiz Durak H. (2019). Examining the acceptance and use of online social networks by preservice teachers within the context of unified theory of acceptance and use of technology model. J. Comput. High. Educ..

[57] Bandura A. (1986). The explanatory and predictive scope of self-efficacy theory. J. Soc. Clin. Psychol..

[58] Meuter M.L., Ostrom A.L., Bitner M.J., Roundtree R. (2003). The influence of technology anxiety on consumer use and experiences with self-service technologies. J. Bus. Res..

[59] Dwivedi Y.K., Rana N.P., Chen H., Williams M.D. A Meta-analysis of the Unified Theory of Acceptance and Use of Technology (UTAUT). Proceedings of the Governance and Sustainability in Information Systems. Managing the Transfer and Diffusion of IT: IFIP WG 8.6 International Working Conference.

[60] Wang Y.-Y., Wang Y.-S. (2022). Development and validation of an artificial intelligence anxiety scale: An initial application in predicting motivated learning behavior. Interact. Learn. Environ..

[61] Şahin F., Şahin Y.L. (2022). Drivers of technology adoption during the COVID-19 pandemic: The motivational role of psychological needs and emotions for pre-service teachers. Soc. Psychol. Educ..

[62] Gunasinghe A., Abd Hamid J., Khatibi A., Azam S.F. (2019). Does anxiety impede VLE adoption intentions of state university lecturers?-a study based on modified UTAUT framework. Eur. J. Soc. Sci. Stud..

[63] Huang L. Acceptance of mobile learning in classroom instruction among college English teachers in China using an extended TAM. Proceedings of the 2017 International Conference of Educational Innovation through Technology (EITT).

[64] Alkhuwaylidee A.R. (2019). Extended unified theory acceptance and use technology (utaut) for e-learning. J. Comput. Theor. Nanosci..

[65] Alyoussef I.Y. (2022). Acceptance of a flipped classroom to improve university students’ learning: An empirical study on the TAM model and the unified theory of acceptance and use of technology (UTAUT). Heliyon.

[66] Peng D. Mobile-based teacher professional training: Influence factor of technology acceptance. Proceedings of the Foundations and Trends in Smart Learning: Proceedings of 2019 International Conference on Smart Learning Environments.

[67] Piniel K. (2013). L2 motivation, anxiety and self-efficacy: The interrelationship of individual variables in the secondary school context. Stud. Second. Lang. Learn. Teach..

[68] Mello R.F., Freitas E., Pereira F.D., Cabral L., Tedesco P., Ramalho G. (2023). Education in the age of Generative AI: Context and Recent Developments. arXiv.

[69] Mishra P., Warr M., Islam R. (2023). TPACK in the age of ChatGPT and Generative AI. J. Digit. Learn. Teach. Educ..

[70] Koh J.H.L., Chai C.S., Tsai C.-C. (2013). Examining practicing teachers’ perceptions of technological pedagogical content knowledge (TPACK) pathways: A structural equation modeling approach. Instr. Sci..

[71] Shulman L.S. (1986). Those who understand: Knowledge growth in teaching. Educ. Res..

[72] Mishra P., Koehler M.J. (2006). Technological pedagogical content knowledge: A framework for teacher knowledge. Teach. Coll. Rec..

[73] Koehler M.J., Mishra P., Kereluik K., Shin T.S., Graham C.R. (2014). The technological pedagogical content knowledge framework. Handbook of Research on Educational Communications and Technology.

[74] Bardakcı S., Alkan M.F. (2019). Investigation of Turkish preservice teachers’ intentions to use IWB in terms of technological and pedagogical aspects. Educ. Inf. Technol..

[75] Teo T., Zhou M. (2017). The influence of teachers’ conceptions of teaching and learning on their technology acceptance. Interact. Learn. Environ..

[76] Mohammad-Salehi B., Vaez-Dalili M., Heidari Tabrizi H. (2021). Investigating Factors That Influence EFL Teachers’ Adoption of Web 2.0 Technologies: Evidence from Applying the UTAUT and TPACK. TESL-EJ.

[77] Lai Wah L., Hashim H. (2021). Determining pre-service teachers’ intention of using technology for teaching english as a second language (Esl). Sustainability.

[78] Yang J., Wang Q., Wang J., Huang M., Ma Y. (2021). A study of K-12 teachers’ TPACK on the technology acceptance of E-schoolbag. Interact. Learn. Environ..

[79] Bandura A., Ramachaudran V.S. (1994). Self-efficacy. Encyclopedia of Human Behavior.

[80] Bandura A. (2006). Guide for constructing self-efficacy scales. Self-Effic. Beliefs Adolesc..

[81] Kinzie M.B. (1990). Requirements and benefits of effective interactive instruction: Learner control, self-regulation, and continuing motivation. Educ. Technol. Res. Dev..

[82] Semiatin A.M., O’Connor M.K. (2012). The relationship between self-efficacy and positive aspects of caregiving in Alzheimer’s disease caregivers. Aging Ment. Health.

[83] Chang C.-T., Hajiyev J., Su C.-R. (2017). Examining the students’ behavioral intention to use e-learning in Azerbaijan? The general extended technology acceptance model for e-learning approach. Comput. Educ..

[84] Zhong Z., Feng S., Jin S. (2023). Investigating the influencing factors of teaching anxiety in Virtual Reality environments. Education and Information Technologies.

[85] Bandura A., Berman P., McLaughlin M., Berman P., McLaughlin M.W. (1997). Self-Efficacy: The Exercise of Control.

[86] Gomez F.C., Trespalacios J., Hsu Y.-C., Yang D. (2022). Exploring teachers’ technology integration self-efficacy through the 2017 ISTE Standards. TechTrends.

[87] Xie T., Zheng L., Liu G., Liu L. (2022). Exploring structural relations among computer self-efficacy, perceived immersion, and intention to use virtual reality training systems. Virtual Real..

[88] Kao C.-P., Tsai C.-C., Shih M. (2014). Development of a survey to measure self-efficacy and attitudes toward web-based professional development among elementary school teachers. J. Educ. Technol. Soc..

[89] Althunibat A. (2015). Determining the factors influencing students’ intention to use m-learning in Jordan higher education. Comput. Hum. Behav..

[90] Altalhi M. (2021). Toward a model for acceptance of MOOCs in higher education: The modified UTAUT model for Saudi Arabia. Educ. Inf. Technol..

[91] Chen H.-R., Tseng H.-F. (2012). Factors that influence acceptance of web-based e-learning systems for the in-service education of junior high school teachers in Taiwan. Eval. Program Plan..

[92] Paraskeva F., Bouta H., Papagianni A. (2008). Individual characteristics and computer self-efficacy in secondary education teachers to integrate technology in educational practice. Comput. Educ..

[93] Wang L., Ertmer P.A., Newby T.J. (2004). Increasing preservice teachers’ self-efficacy beliefs for technology integration. J. Res. Technol. Educ..

[94] Tondeur J., Aesaert K., Prestridge S., Consuegra E. (2018). A multilevel analysis of what matters in the training of pre-service teacher’s ICT competencies. Comput. Educ..

[95] Wang Q., Zhao G. (2021). ICT self-efficacy mediates most effects of university ICT support on preservice teachers’ TPACK: Evidence from three normal universities in China. Br. J. Educ. Technol..

[96] Brislin R.W. (1970). Back-translation for cross-cultural research. J. Cross-Cult. Psychol..

[97] Dong Y., Xu C., Chai C.S., Zhai X. (2020). Exploring the structural relationship among teachers’ technostress, technological pedagogical content knowledge (TPACK), computer self-efficacy and school support. Asia-Pac. Educ. Res..

[98] Celik I. (2023). Towards Intelligent-TPACK: An empirical study on teachers’ professional knowledge to ethically integrate artificial intelligence (AI)-based tools into education. Comput. Hum. Behav..

[99] Noar S.M. (2003). The role of structural equation modeling in scale development. Struct. Equ. Model..

[100] Fornell C., Larcker D.F. (1981). Evaluating structural equation models with unobservable variables and measurement error. J. Mark. Res..

[101] Hair J.F., Hult G.T.M., Ringle C.M., Sarstedt M. (2017). A Primer on Partial Least Squares Structural Equation Modeling (PLS-SEM). Int. J. Res. Method Educ..

[102] Hair J.F., Ringle C.M., Sarstedt M. (2011). PLS-SEM: Indeed a silver bullet. J. Mark. Theory Pract..

[103] Hair J.F., Black W.C., Babin B.J., Anderson R.E. (2014). Multivariate Data Analysis: Pearson New International Edition.

[104] Dečman M. (2015). Modeling the acceptance of e-learning in mandatory environments of higher education: The influence of previous education and gender. Comput. Hum. Behav..

[105] Al-Shehri M. (2017). The effectiveness of D2L system: An evaluation of teaching-learning process in the Kingdom of Saudi Arabia. Int. J. Adv. Comput. Sci. Appl..

[106] Hu S., Laxman K., Lee K. (2020). Exploring factors affecting academics’ adoption of emerging mobile technologies—An extended UTAUT perspective. Educ. Inf. Technol..

[107] Li Q., Liu Q., Chen Y. (2023). Prospective Teachers’ Acceptance of virtual reality technology: A mixed study in Rural China. Educ. Inf. Technol..

[108] Al-Adwan A.S., Yaseen H., Alsoud A., Abousweilem F., Al-Rahmi W.M. (2022). Novel extension of the UTAUT model to understand continued usage intention of learning management systems: The role of learning tradition. Educ. Inf. Technol..

[109] Wang W.-T., Wang C.-C. (2009). An empirical study of instructor adoption of web-based learning systems. Comput. Educ..

[110] Ni A., Cheung A. (2023). Understanding secondary students’ continuance intention to adopt AI-powered intelligent tutoring system for English learning. Educ. Inf. Technol..

[111] Jung I., Lee Y. (2015). YouTube acceptance by university educators and students: A cross-cultural perspective. Innov. Educ. Teach. Int..

[112] Almisad B., Alsalim M. (2020). Kuwaiti female university students’ acceptance of the integration of smartphones in their learning: An investigation guided by a modified version of the unified theory of acceptance and use of technology (UTAUT). Int. J. Technol. Enhanc. Learn..

[113] Kamalasena B., Sirisena A. (2023). Factors Affecting Postgraduate Student’s Intention in Using an E-examination System During the COVID-19 Pandemic: Application of UTAUT Model. South Asian J. Bus. Insight.

[114] Holzmann P., Schwarz E.J., Audretsch D.B. (2020). Understanding the determinants of novel technology adoption among teachers: The case of 3D printing. J. Technol. Transf..

[115] Green B.P. (2020). Artificial Intelligence and Ethics: Sixteen Challenges and Opportunities. Markkula Center for Applied Ethics at Santa Clara University. https://www.scu.edu/ethics/all-about-ethics/artificial-intelligence-and-ethics-sixteen-challenges-and-opportunities.

[116] Rahmiati F., Jelitalia A. (2021). Extending the Role of Technology Acceptance Model (TAM) with Perceived Risk and E-Customer Service. J. Technol. Manag. Technopreneurship (JTMT).

[117] Jang J., Ko Y., Shin W.S., Han I. (2021). Augmented reality and virtual reality for learning: An examination using an extended technology acceptance model. IEEE Access.

[118] Ertmer P.A., Ottenbreit-Leftwich A.T., Sadik O., Sendurur E., Sendurur P. (2012). Teacher beliefs and technology integration practices: A critical relationship. Comput. Educ..

[119] Joo Y.J., Park S., Lim E. (2018). Factors influencing preservice teachers’ intention to use technology: TPACK, teacher self-efficacy, and technology acceptance model. J. Educ. Technol. Soc..

[120] Kim B., Park M.J. (2018). Effect of personal factors to use ICTs on e-learning adoption: Comparison between learner and instructor in developing countries. Inf. Technol. Dev..

[121] Wong K.-T., Muhammad M., Abdullah N. (2020). Exploring the drivers of intention to use interactive whiteboards among Malaysia university students: Does technology self-efficacy matter?. Int. J. Emerg. Technol. Learn. (Ijet).

[122] Rahmawati R.N. (2019). Self-efficacy and use of e-learning: A theoretical review technology acceptance model (TAM). Am. J. Humanit. Soc. Sci. Res..

[123] Putry A.R.A., Astuti P., Sakhiyya Z. (2022). The manifestation of EFL teachers’ self-efficacy and TPACK with their teaching performance. Engl. Educ. J..

[124] Kul U., Aksu Z., Birisci S. (2019). The Relationship between Technological Pedagogical Content Knowledge and Web 2.0 Self-Efficacy Beliefs. Online Submiss..

[125] Olivier T.A., Shapiro F. (1993). Self-efficacy and computers. J. Comput.-Based Instr..

[126] Hong J.-C., Cao W., Liu X., Tai K.-H., Zhao L. (2023). Personality traits predict the effects of Internet and academic self-efficacy on practical performance anxiety in online learning under the COVID-19 lockdown. J. Res. Technol. Educ..

[127] Alnoor A.M., Al-Abrrow H., Abdullah H., Abbas S. (2020). The impact of self-efficacy on employees’ ability to accept new technology in an Iraqi university. Glob. Bus. Organ. Excell..

[128] Sánchez-Prieto J.C., Olmos-Migueláñez S., García-Peñalvo F.J. (2017). MLearning and pre-service teachers: An assessment of the behavioral intention using an expanded TAM model. Comput. Hum. Behav..

[129] Fernández-Batanero J.-M., Román-Graván P., Reyes-Rebollo M.-M., Montenegro-Rueda M. (2021). Impact of educational technology on teacher stress and anxiety: A literature review. Int. J. Environ. Res. Public Health.

[130] Jena R. (2015). Technostress in ICT enabled collaborative learning environment: An empirical study among Indian academician. Comput. Hum. Behav..

[131] Venkatesh V., Davis F.D. (1996). A model of the antecedents of perceived ease of use: Development and test. Decis. Sci..

[132] Obienu A.C., Amadin F.I. (2021). User acceptance of learning innovation: A structural equation modelling based on the GUAM framework. Educ. Inf. Technol..

[133] Alasmari T., Zhang K. (2019). Mobile learning technology acceptance in Saudi Arabian higher education: An extended framework and A mixed-method study. Educ. Inf. Technol..

[134] NYAABA M., Xiaoming Z. (2024). Generative AI professional development needs for teacher educators. J. AI.

[135] Kapici H.O., Akcay H. (2023). Improving student teachers’ TPACK self-efficacy through lesson planning practice in the virtual platform. Educ. Stud..

[136] Liou Y.-H., Daly A.J., Canrinus E.T., Forbes C.A., Moolenaar N.M., Cornelissen F., Van Lare M., Hsiao J. (2017). Mapping the social side of pre-service teachers: Connecting closeness, trust, and efficacy with performance. Teach. Teach..

[137] Sidik D., Syafar F. (2020). Exploring the factors influencing student’s intention to use mobile learning in Indonesia higher education. Educ. Inf. Technol..

